# Effects of iguratimod on inflammatory factors and apoptosis of submandibular gland epithelial cells in NOD mice

**DOI:** 10.1038/s41598-023-45529-x

**Published:** 2023-10-24

**Authors:** Shuying Wang, Jiake Yu, Jie Yang, Yan Ge, Jing Tian

**Affiliations:** 1 Affiliated Nanhua Hospital, University of South China, Hengyang, 421000 Hunan China; 2 Department of Rheumatology and Immunology, The 2nd Xiangya Hospital of Central South University, Changsha, 410000 Hunan China

**Keywords:** Rheumatology, Rheumatic diseases

## Abstract

Non-obese diabetic (NOD) mice were taken as primary Sjögren’s syndrome (pSS) model mice to examine the therapeutic impact of iguratimod (IGU) on inflammatory factors levels and apoptosis of submandibular epithelial cells, and provide experimental basis for the treatment of pSS by iguratimod. Twenty-four NOD murine models were divided into the model, high-dose (IGU 30 mg/kg) and low-dose (IGU 10 mg/kg) groups, eight mice per group. The normal control group comprised eight C57B/L mice. From 8 weeks of age, the NOD mice were administered IGU by intragastric gavage administration every day for 8 weeks; their water consumption, saliva secretion, submandibular gland, and spleen indices were measured. The levels of serum inflammatory factor (IL-1β, TNF-α, IL-6, and IL-17) were evaluated, and Bax, caspase-3, and Bcl-2 levels were detected. The histological alterations in the submandibular glands were discovered. IGU can reduce the water intake of NOD mice (*p* < 0.01), increase the saliva secretion and the submandibular gland index (*p* < 0.01); reduce the spleen index and the serum inflammatory factors (*p* < 0.01); improve the pathological tissue damage and cell apoptosis of the submandibular gland (*p* < 0.05). IGU can reduce the expression levels of inflammatory mediators in the serum and the extent of lymphocyte infiltration and apoptosis in submandibular gland epithelial cells. It can also regulate apoptosis-related protein expression, thereby improving the secretory function of exocrine glands.

## Introduction

PSS is a chronic and gradually progressive autoimmune disease characterized by salivary and lacrimal glands dysfunction and destruction, accompanied by extensive systemic manifestations^1^. The main characteristics of Sjögren’s syndrome(SS) include dry eyes and mouth, fatigue, musculoskeletal pain, and major salivary gland swelling^2^. Studies have shown that T and B lymphocytes, cytokines [including tumor necrosis factor (TNF), interleukins (ILs), and B-cell activating factors (BAFFs), and autoantibodies are involved in SS pathogenesis^3,4^. Furthermore, salivary gland epithelial cell apoptosis has been shown to cause gland atrophy and secretory dysfunction^5,6^.

Iguratimod (IGU) is a new anti-rheumatic drug mainly used in China and Japan to treat rheumatoid arthritis (RA)^7–9^. It can suppress the secretion of IL-1, IL-6, IL-8, IL-17, TNF-α, interferon-gamma (IFN-γ), and other inflammatory cytokines, as well as secretion from mononuclear cells and synovial fibroblasts. It directly acts on B lymphocytes and inhibits immunoglobulin (Ig) production. IGU can also selectively inhibit cyclo-oxygenase (COX)-2 enzyme activity and downregulate the nuclear factor (NF)-κB signaling pathway to reduce bone destruction^10–12^. Currently, doctors in China have begun using IGU to treat SS as well. In patients with pSS, IGU can effectively reduce erythrocyte sedimentation rate, rheumatoid factor and IgG levels, platelet counts, and other laboratory indicators alleviating dryness of mouth and eyes experienced by patients^13^. IGU has also been proven to improve the disease activity indices as well as patient-reported indices of the European League Against Rheumatism (EULAR)^14–16^. Therefore, IGU is an effective and safe drug for SS; however, its mechanism of action remains unclear to date.

The SS non-obese diabetic (NOD) model is a murine model with spontaneously-developed type 1 diabetes. It was first developed by Japanese scientists. Interestingly, in the NOD mice’s salivary and lacrimal glands, researchers discovered remarkably elevated inflammatory cell infiltration levels, comparable to that reported in pSS. In addition, many pSS-related autoantibodies were found in the serum of NOD mice, including anti-muscarinic type 3 acetylcholine receptors, anti-120-kDa α-fodrin, anti-La/SSB, and anti-Ro/SSB antibodies^17^.

The purpose of this study is to explore the therapeutic effect of IGU and its influence on inflammatory factors and cell apoptosis to provide reliable experimental data for the treatment of SS.

## Materials and methods

### Statement

The animal use protocol listed below has been reviewed and approved by the Institutional Animal Care and Use Committee (IACUC), The Second Xiangya Hospital, Central South University, China. All methods were carried out in accordance with relevant guidelines and regulations. All methods are reported in accordance with ARRIVE guidelines.

### Animals

Twenty-four female, 8-week-old NOD mice and eight C57BL/6 mice, whose weights were between 18–20 g, were acquired from Changzhou Cavins Laboratory Animal Co., Ltd. (Changzhou, China). We categorized the NOD mice at random into the model, high-dose IGU (IGU 30 mg/kg) and low-dose IGU (IGU 10 mg/kg) groups, eight mice per group. The normal control group comprised the C57B/L mice. The mice were raised in a controlled setting with a consistent temperature, humidity, and atmosphere, and were supplied with water in drinking containers. By taking eyeball bloodletting, the mouse blood were collected and the mouse were euthanized by dislocation of the cervical spine under deep anesthesia.

### IGU treatment

IGU was procured from Simcere Pharmaceutical Research Co., Ltd. (Jiangsu, China). Before in vivo administration of the drug, it was first suspended in 0.5% methylcellulose solution. At 8 weeks of age (experiment day 0), 10 and 30 mg/kg IGU were administered to the low- and high-dose IGU groups by intragastric gavage administration, once a day at a fixed time point. Each mouse in the model control and normal control groups was given an equal volume of 0.5% carboxymethylcellulose by intragastric gavage administration, once a day at a fixed time point.

### Measurement of water intake

The water intake of mice was measured in everyday of the experiment using the following method: recorded the daily water intake of each group of mice, prepared drinking water for mice daily at 9:00 am, recorded the amount of water as W0 and record the remaining water at 9:00 am the next day as W1. The water intake was equal to W0–W1 (ml). Finally, the average daily water intake of each group of mice was calculated.

### Measurement of salivary flow rate

The salivary flow of mice was measured in the fifth, sixth, seventh, and eighth weeks of the experiment using the following method: first, the dry weight of a small cotton ball was measured on an electronic balance, recorded as M0. Then, The mice were anesthetized by injected intraperitoneally with pentobarbital sodium (50 mg/kg; Merck KgaA, Germany). After the mice were fully anesthetized, the small cotton ball was placed in the mouth of the mice near the submandibular gland duct, removed after 5 min, and weighed again. This weight was recorded as M1, and the salivary flow rate of the mice was calculated as M1–M0 (mg/5 min), saliva was stored at − 70 °C until analysis^18^.

### Determination of submandibular gland and spleen indices

The mice were weighed and euthanized on the eighth weekend of the experiment. The submandibular gland and spleen were removed and weight measurements were taken. The weight of each organ was calculated as the ratio of the weight of each organ to the total body weight.

### Enzyme-linked immunosorbent assay (ELISA)

At the eighth week of the experiment, blood samples were collected from each mouse via a retro-orbital puncture shortly before euthanizing them. The plasma was isolated by 15 min of centrifugation at 1000×*g* at ambient temperature. Next, IL-17, IL-6, TNF-α, and IL-1β serum levels were determined utilizing commercial ELISA kits (Elabscience Biotechnology, Wuhan, China) in accordance with the guidelines stipulated by the manufacturer.

### Western blotting

Protein specimens were loaded onto sodium dodecyl-sulfate (SDS)-polyacrylamide precast gels, transferred to polyvinylidene difluoride membranes, and subjected to incubation with primary antibodies throughout the night. This was followed by an additional incubation with a secondary antibody in a similar manner. BandScan 5.0 program (Glyko, Novato, CA, USA) was employed to measure the grey value of each band, and the endogenous control used was glyceraldehyde-3-phosphate dehydrogenase (GAPDH). The primary antibodies included rabbit anti-GAPDH (AB-P-R 001, 1:1000, Hangzhou Goodhere), mouse anti-Bax (60267-1-ig, 1:1000, Proteintech), rabbit anti-Bcl-2 (26593-1-ap, 1:1000, Proteintech), and mouse anti-caspase-3 (66470-2-AP, 1:1000, Proteintech). The secondary antibodies used included rabbit anti-mouse IgG H&L (HRP; BA1058, 1:1000, Boster) and rabbit anti-goat IgG H&L (HRP; BA1060, 1:1000, Boster).

### Terminal deoxynucleotidyl transferase dUTP nick-end labeling (TUNEL)

The slides were treated with a 1:9 mixture of reagent 1 (TdT) and reagent 2 (dUTP) (TUNEL kit; Roche; 11684817910). Phosphate-buffered saline (PBS; pH 7.4) was used to soak the slides. Next, DAPI staining solution was added dropwise, followed by incubation of the slides at ambient temperature in darkness. Anti-fluorescence quenching mounting plates were used to mount the slides, which were then examined with the aid of a fluorescence microscope (Nikon, Japan). The images were captured, and the fluorescence intensity was assessed utilizing the BandScan 5.0 software.

### Histological analysis

The glands in the submandibular glands were fixed in 10 percent neutral formalin before embedding them in paraffin and cutting them into 5 µm slices. Haematoxylin and eosin (HE) staining was used, and a histomorphological investigation was performed on the slices using a light microscope. The samples were scored according to the Chisholm–Mason scale^19^ as follows: 0, occasional lymphocytic infiltration; 1, a few lymphocytic infiltrates that are scattered; 2, moderate lymphocytic infiltration (no lesions) with moderate parenchymal injury; 3, occasional 0–1 lymphocytic infiltration lesion/five low power fields with moderate parenchymal injury; and 4, about 2–3 lymphocytic infiltration lesions/five low power fields with severe parenchymal injury^20^.

### Statistical analyses

Analyses of statistical data were accomplished utilizing GraphPad Prism (version 9 for Windows; GraphPad Software, San Diego, CA, USA). The data are reported as mean ± standard deviation (SD). For group comparisons, we utilized a one-way analysis of variance. p < 0.05 was established as the criterion of statistical significance.

## Results

### The effect of IGU on the general condition of NOD mice

**Figure 1:**
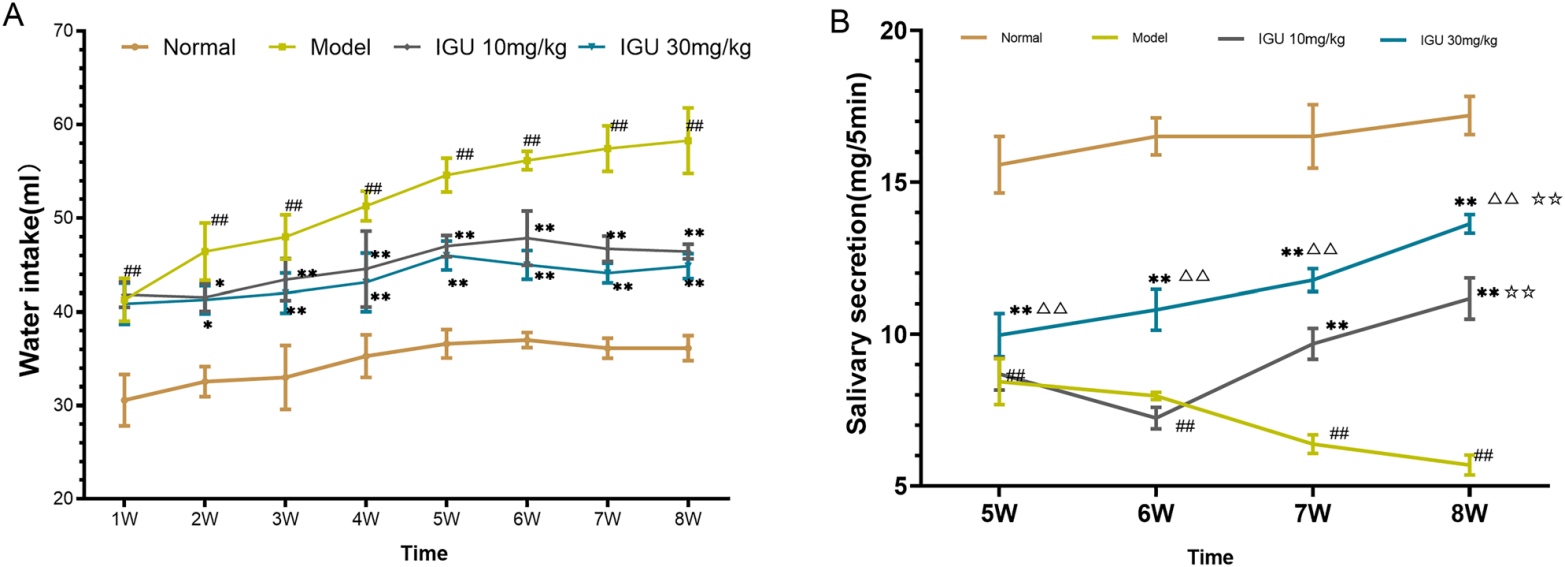
Impacts of iguratimod (IGU) on water intake (**A**) and salivary secretion (**B**) in non-obese diabetic (NOD) mice. Model vs. normal, ^#^*p* < 0.05, ^##^*p* < 0.01. IGU 10 mg/kg and IGU 30 mg/kg vs. model, *p < 0.05, **p < 0.01. IGU 30 mg/kg vs. IGU 10 mg/kg, ^△^*p* < 0.05, ^△△^*p* < 0.01.

Throughout the experiment, the normal control group consumed lesser water than the other groups. However, the model control group consistently maintained a higher water consumption level, with the values at each time point being greater as opposed to those of the normal control group (*p* < 0.01). The water intake of the IGU groups (10- and 30-mg/kg IGU) was similar to that of the model group during the first week and lesser than the model group from the 2nd week (*p* < 0.05). From the sixth week of the experiment, the water intake of mice in the low- and high-dose IGU groups began to decrease, suggesting that IGU alleviated the need for increased water consumption among NOD mice (Fig. 1A) (Supplementary File 1 is about water intake).

Mice in the normal group maintained a high level of salivary secretion, and no significant change was noted over time. The salivary secretion of the model group mice decreased as the experiment progressed, and was considerably reduced as opposed to that in the low- and high-dose groups at weeks five and six (both *p* < 0.01). The salivary secretion of the IGU groups increased with the length of the intervention period, and the secretion level of the high-dose group was remarkably elevated as opposed to that of the low-dose group (*p* < 0.01) (Fig. 1B).

**Figure 2:**
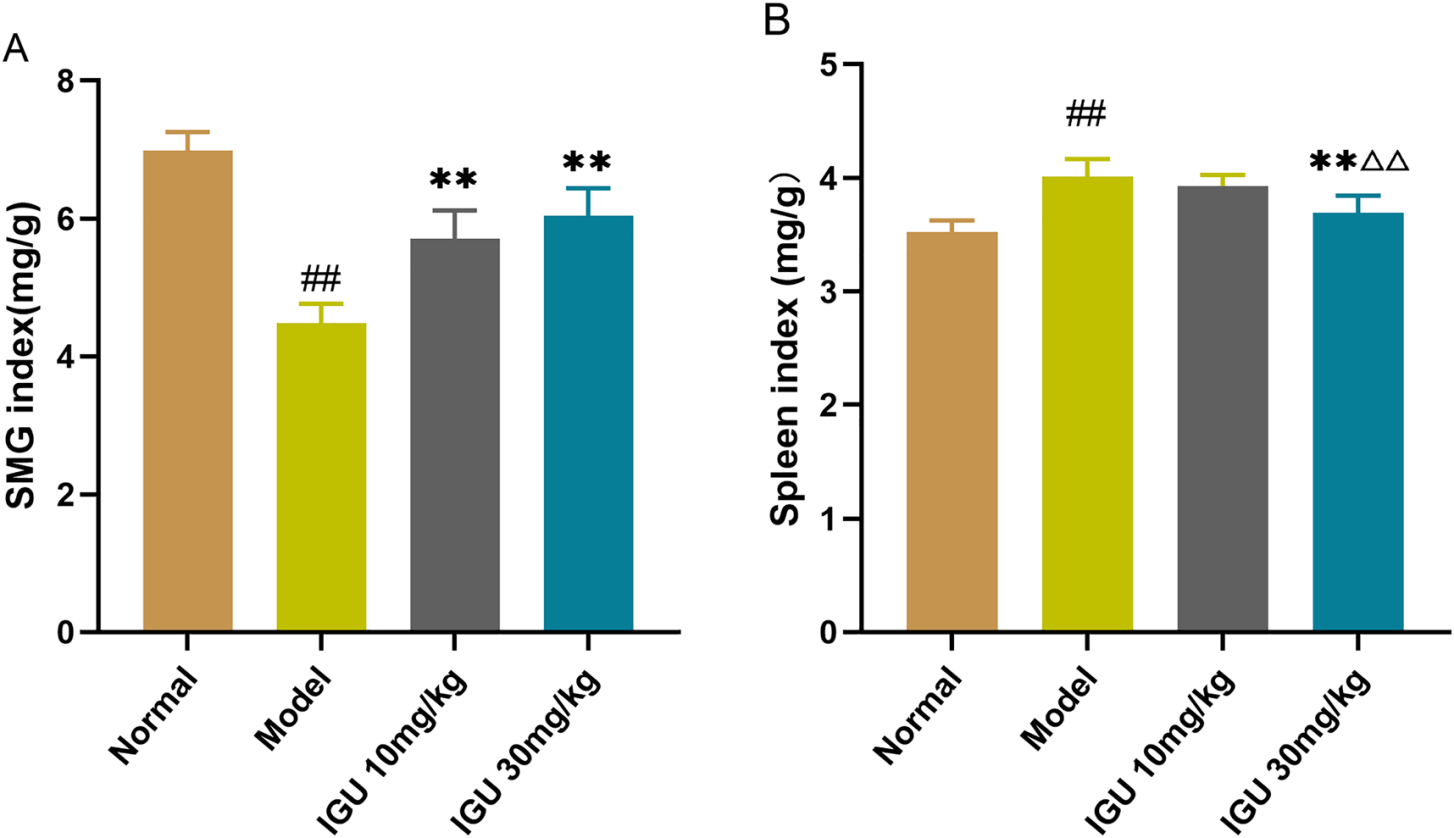
Impacts of iguratimod (IGU) on submandibular gland (**A**) and spleen (**B**) indices in non-obese diabetic (NOD) mice. Model vs. normal, ^#^*p* < 0.05, ^##^*p* < 0.01. IGU 10 mg/kg and IGU 30 mg/kg vs. model, *p < 0.05, **p < 0.01. IGU 30 mg/kg vs. IGU 10 mg/kg, ^△^*p* < 0.05, ^△△^*p* < 0.01. IGU 10 mg/kg (8 weeks) vs. IGU 10 mg/kg (5 weeks). IGU 30 mg/kg (8 weeks) vs. IGU 30 mg/kg (5 weeks), ^✫^*p* < 0.05, ^✫✫^*p* < 0.01.

The submandibular index of the normal group was considerably elevated as opposed to that of the model control group (*p* < 0.01). After IGU administration, the submandibular index of the normal group mice increased remarkably compared with the model control group. The spleen index of the normal group mice was substantially lowered as opposed to that of the model group (*p* < 0.01) (*p* < 0.01; Fig. 2).

### Impact of IGU on serum inflammatory factor levels in NOD mice

**Figure 3:**
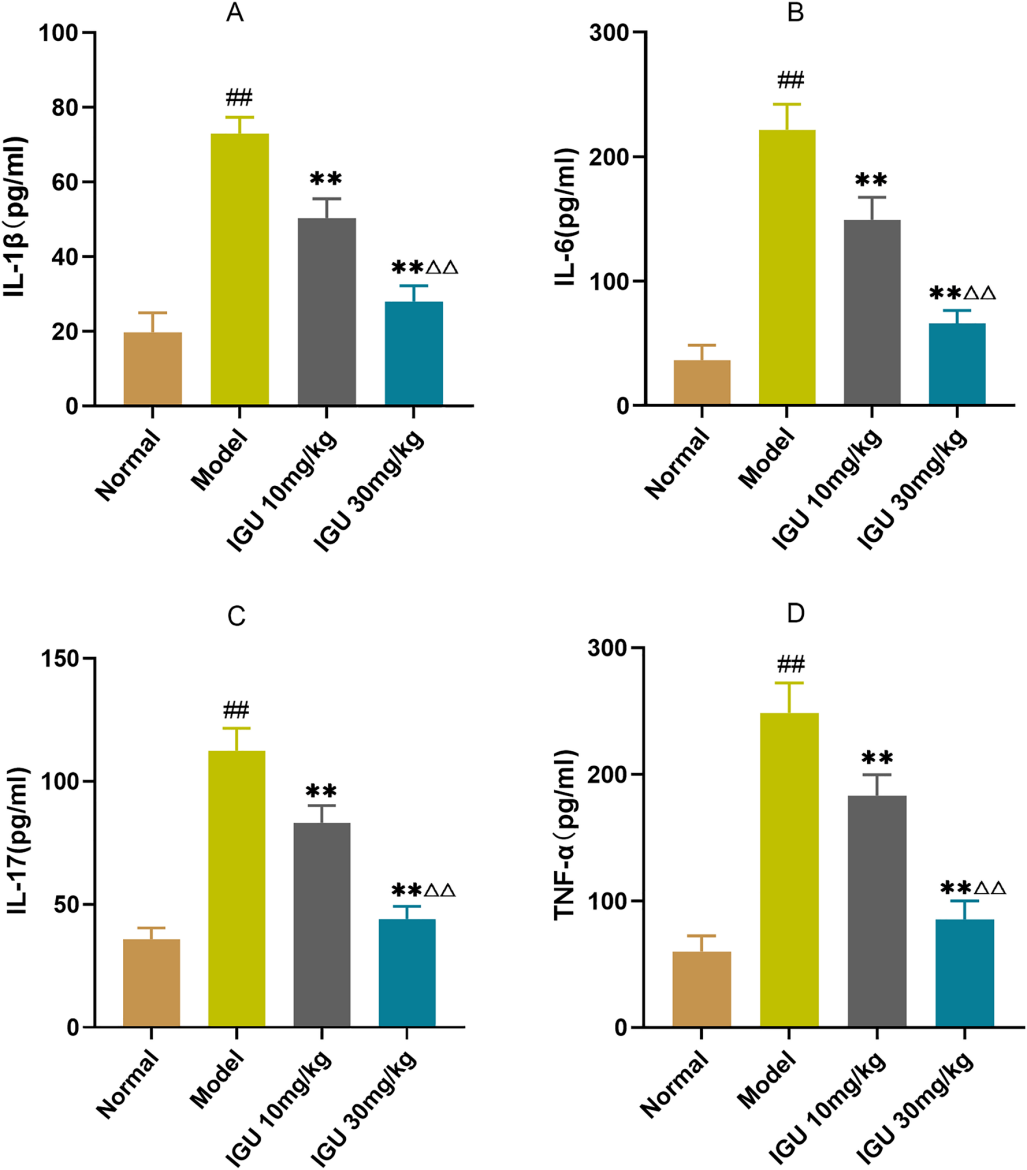
Impacts of iguratimod (IGU) on inflammatory factor levels (IL-6, TNF-α, IL-17, and IL-1β) in non-obese diabetic (NOD) mice. Model vs. normal, ^#^*p* < 0.05, ^##^*p* < 0.01. IGU 10 mg/kg and IGU 30 mg/kg vs. model, *p < 0.05, **p < 0.01. IGU 30 mg/kg vs. IGU 10 mg/kg, ^△^*p* < 0.05, ^△△^*p* < 0.01.

The normal group expressed the lowest levels of serum inflammatory markers: IL-6, TNF-α, IL-17, and IL-1β, while the model group expressed the highest levels. After administering different IGU doses, the expression levels of serum inflammatory mediators IL-6, TNF-α, IL-17, and IL-1β gradually decreased. However, they were considerably elevated in the high-dose group in contrast with the low-dose group (*p* < 0.01) (Fig. 3).

### Impacts of IGU on apoptosis in submandibular gland cells in NOD mice

**Figure 4:**
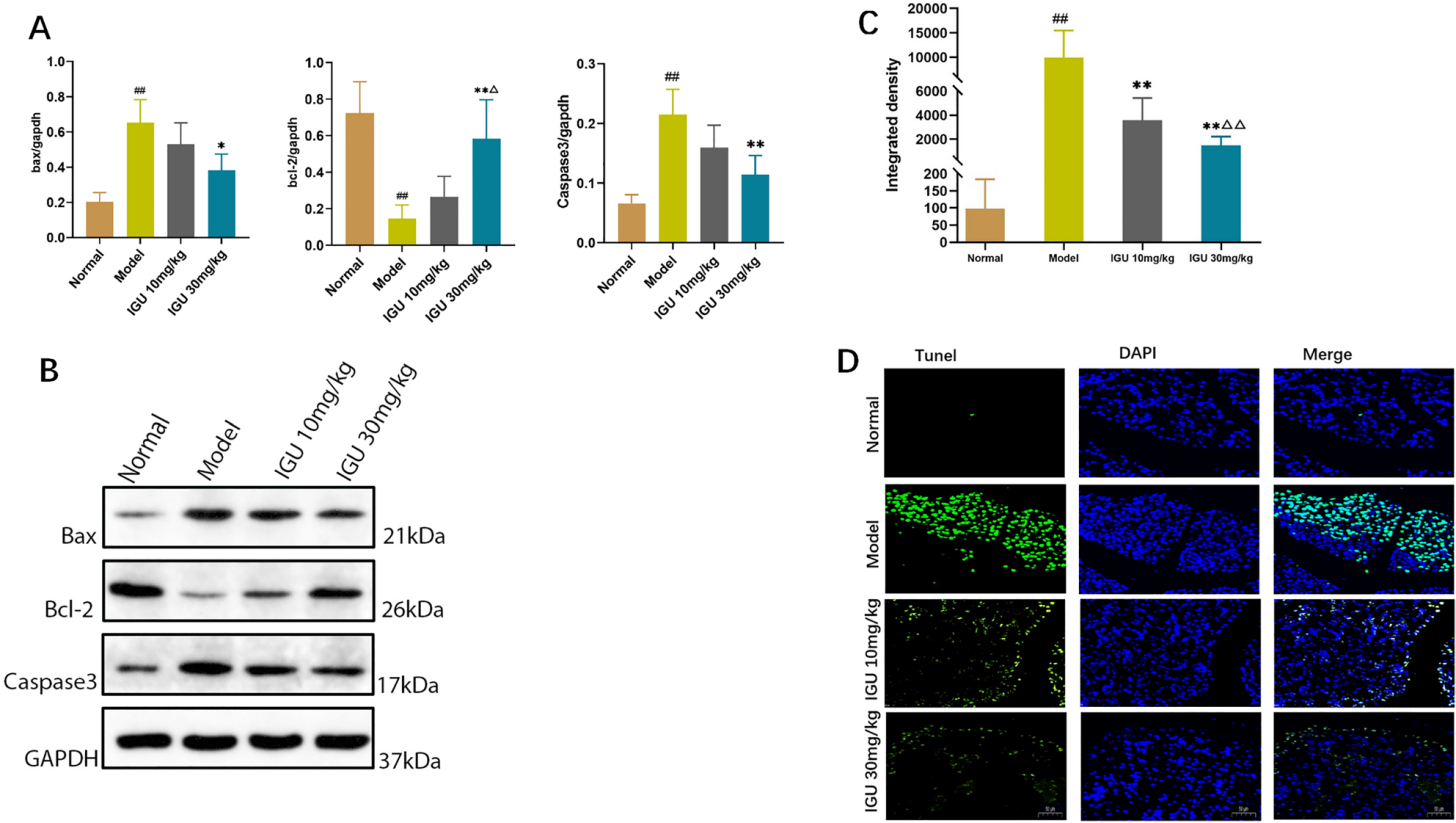
Effect of iguratimod (IGU) on the levels of apoptosis-related proteins (Bcl-2, Caspase-3 and Bax) in non-obese diabetic (NOD) mice (**A**). Model vs. normal, ^#^*p* < 0.05, ^##^*p* < 0.01. IGU 10 mg/kg and IGU 30 mg/kg vs. model, *p < 0.05, **p < 0.01. IGU 30 mg/kg vs. IGU 10 mg/kg, ^△^*p* < 0.05, ^△△^*p* < 0.01. Western blotting analysis was utilized to evaluate the expression levels of apoptosis-associated proteins (**B**). Effect of iguratimod (IGU) on apoptosis in submandibular gland epithelial cells in non-obese diabetic (NOD) mice (**C**,**D**).

The normal and model group NOD mice had the lowest and highest submandibular gland levels of Bax and caspase-3 expression, respectively. Bax and cleaved caspase-3 expression gradually decreased after delivering different IGU doses, unlike that of Bcl-2 (Fig. 4A,B) (Supplementary File 2 is about better resolution for Western blot results).

The normal and model groups displayed the lowest and highest apoptosis levels, respectively, in the submandibular gland epithelial cells. Apoptosis rates gradually improved after administering different IGU doses (Fig. 4C,D).

### Effect of IGU on lymphocyte infiltration

**Figure 5:**
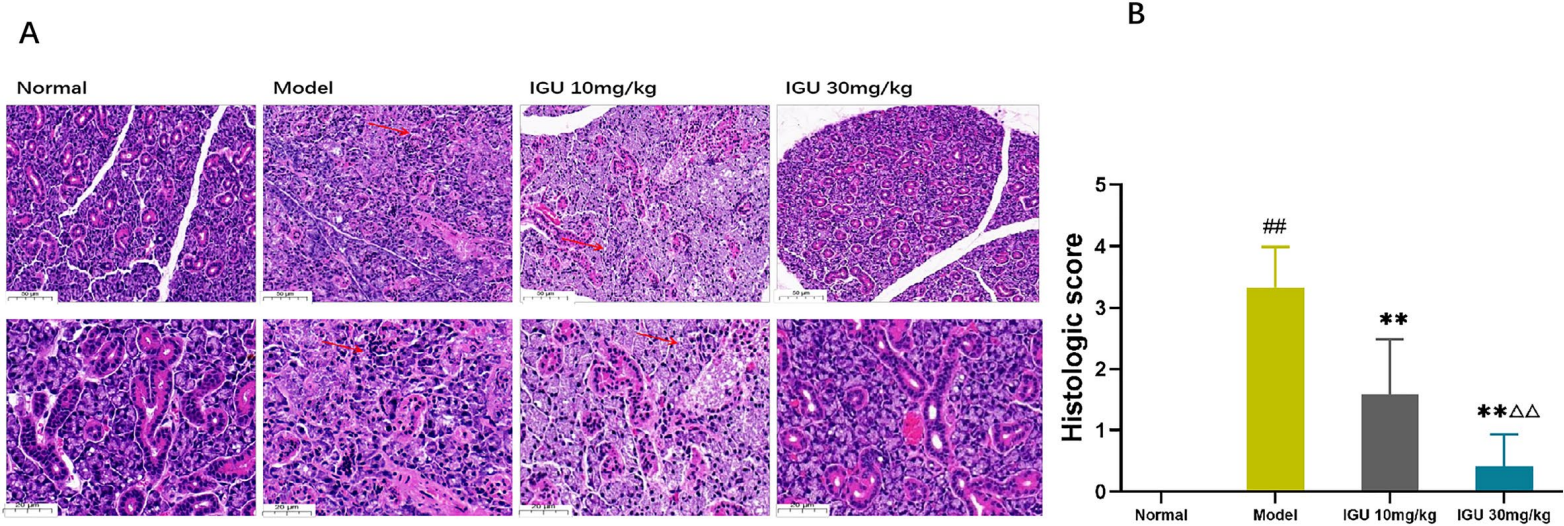
Effect of IGU on lymphocyte infiltration. Salivary gland tissue sections stained with HE (**A**). Histopathological evaluation as per the Cutler’s method and the calculated pathological scores (**B**). Red arrows indicate lymphocyte infiltration and gland destruction. Model vs. normal, ^#^*p* < 0.05, ^##^*p* < 0.01. IGU 10 mg/kg and IGU 30 mg/kg vs. model, *p < 0.05, **p < 0.01. IGU 30 mg/kg vs. IGU 10 mg/kg, ^△^*p* < 0.05, ^△△^*p* < 0.01.

The normal mice's submandibular glands were lobulated and had few lymphocytes. The acini were uniform in size and tightly arranged. The inner wall of the gland was smooth, without any atrophy or expansion, and no vasodilation or fibrosis was observed. In the model group, the acini of different shapes and sizes were infiltrated by lymphocytes and appeared in small numbers. In comparison to the model group, the submandibular gland cells of the low-dose group showed lesser damage to the glandular parenchyma; the acini of different sizes were destroyed; the lumen was dilated. For the high-dose group, the degree of lymphocyte infiltration and parenchymal damage was lower in comparison to that in the model group. There was a small amount of lymphocyte infiltration, and the acini and glandular duct were nearly equal in size (Fig. 5).

## Discussion

In this study, IGU can reduce the expression levels of inflammatory mediators in the serum and the extent of lymphocyte infiltration and apoptosis in submandibular gland epithelial cells. It can also regulate apoptosis-related protein expression, thereby improving the secretory function of exocrine glands (Supplementary Information).

The pathological changes in the NOD mice’s submandibular glands were congruent with those of SS. At 12–16 weeks of age, a reduction in tears and saliva was observed, and lymphocytes were found to be scattered and infiltrated around the gland ducts. As the disease aggravated, the extent of lymphocyte infiltration gradually increased; the number of acini reduced, and the acinar structure was destroyed^21,22^.

IGU can reduce the expression levels of inflammatory mediators in the serum and the extent of lymphocyte infiltration and apoptosis in submandibular gland epithelial cells. It can also regulate apoptosis-related protein expression, thereby improving the secretory function of exocrine glands. In the study, two doses of 10 mg/kg and 30 mg/kg were selected for experimental research. 10 mg/kg was calculated based on the equivalent dose of humans and mice as a low-dose group^23^. In the MRL/lpr of lupus nephritis model mice, 8-week-old mice were given IGU 30 mg/kg to intervene, and the disease activity markers (anti-dsDNA antibodies and immunoglobulins) in the serum of experimental mice after 21 weeks decreased, improved hypocomplementemia and decreased expression of cytokines such as IL-6, IL-17A and IL-21, and unconspicuous toxic and side effects of IGU were observed, indicating that 30 mg/kg has a good effect on lupus nephritis mouse model^24^. Therefore, 30 mg/kg was selected as the high-dose group.

IGU inhibited submandibular gland lymphocyte infiltration and ameliorated their destruction and atrophy, similar to the effects of total glucosides of paeony in NOD mice^25^. Currently, there is no clinically effective drug for dry mouth. Herein, IGU is a potential drug for these symptoms. The spleen is an important peripheral immune organ for antibody production. When its resident immune cells are stimulated by foreign antigens, they proliferate and differentiate, thereby increasing the volume and mass of the spleen^26^. In this experiment, IGU can increase the submandibular gland index and reduce the spleen index in NOD mice. He Huizhen^27^ experiments showed that Zengye Bujin Decoction can significantly increase the submandibular gland index and significantly reduce the spleen index of mice. It shows that IGU may prevent the destruction and atrophy of the submandibular glands, inhibit the proliferation and activation of immune cells in the spleen, and reduce the immune response in NOD mice.

Serum levels of inflammatory factors in NOD mice significantly decreased after administering different IGU doses, similar to the results obtained with IGU for RA and ankylosing spondylitis^28,29^. IGU can inhibit the IL-1β, TNF-α, IL-17, and IL-6 expressions. Therefore, curtailing inflammatory cytokine release lowers the extent of lymphocyte infiltration and improves disease symptoms. In Yuan's experiment^30^, IGU (10 mg/kg and 30 mg/kg) was given to intervene lupus nephritis mice. It was found that IGU could improve the symptoms of lupus nephritis, attenuate the pathological changes of glomerulonephritis and tubular nephritis, reduce glomerular immune complex deposition, and reduce serum dsDNA level by regulating Th17/Treg ratio in mice in a dose-dependent manner. A study^31^ found that IGU downregulated INF-γ, IL-18, IL-6, and IL-17 expression in the peripheral blood of RA patients and decreased the frequency of Th17 and Th1 cells while significantly increasing the frequency of regulatory T cells (Tregs). The frequency of the subpopulations was consistent, suggesting that IGU may act on T cell subpopulations and affect the secretion of T cell-related cytokines to treat autoimmune diseases. The NOD-like receptor family pyrin domain containing 3 (NLRP3) inflammasome performs a critical function in the adaptive and innate immune system and mediates the occurrence and development of various autoimmune disorders, particularly RA, SS, systemic lupus erythematosus, and ankylosing spondylitis. Activated NLRP3 stimulates pro-caspase-1, triggering IL-1β, a pathway referred to as the NLRP3/IL-1 axis^32^. Studies^33,34^ have shown that IGU can reduce IL-1β levels by inhibiting NLRP3 activation, suggesting that IGU may inhibit NLRP3 and thus downregulate IL-1β levels in pSS. After binding to the receptor, IL-17 activates NF-kB and MAPK in the form of Act1–TRAF6 and Act1–TRAF5, respectively, and further stimulates downstream inflammatory signaling pathways. IGU can specifically disrupt Act1-TRAF5 and Act1-IKKi interactions without affecting Act1-TRAF6 binding^35^ in RA, which explains the inhibitory effect of IGU on the IL-17 pathway.

Apoptosis is a complex process. The Fas–FasL and Bcl-2 protein families regulate apoptosis in salivary gland epithelial cells in pSS patients^6^. Caspase-3 performs an integral function in apoptosis by cleaving proteins such as DNA polymerase, leading to DNA fragmentation, a defining characteristic of apoptotic cell death. Bcl-2 can inhibit mitochondrial disintegration caused by various factors, prevent the release of pro-apoptotic factors such as cyt *c*, and prevent caspase-3 activation^36^. As a signaling pathway that modulates apoptotic cell death and survival, the Bcl-2/Bax/cleaved caspase-3 apoptosis signaling pathway is associated with many diseases. Puerarin has been reported to^37^ reduce the degeneration and apoptosis of cortical neurons by inhibiting the Bcl-2/Bax/cleaved caspase-3 apoptosis signaling pathway. In this experiment, Bax and caspase-3 expression levels in the NOD mice’s submandibular glands were elevated, and Bcl-2 expression was decreased, both of which were consistent with previous reports^36^. Traditional Chinese medicines, such as Xuanfei Bujin granules and Dendrobium Officinale, can reportedly reduce apoptosis in submandibular gland tissues^38,39^. This study revealed that IGU could ameliorate apoptosis in NOD mice’s submandibular gland epithelial cells by regulating Bax and Bcl-2 protein expression.

Previous experimental results have shown that IGU induces the apoptosis of fibroblast-like synoviocytes (FLS) and peripheral blood mononuclear cells in RA^11,40,41^. IGU also induces apoptosis in glioma and multiple myeloma^42,43^. Nevertheless, this study is the first to report that IGU can improve the apoptosis of mouse exocrine glands by inhibiting caspase-3 activation and the apoptosis protein Bcl-2 and pro-apoptosis protein Bax expression. The different results with IGU among patients with RA and tumors may be related to the different roles of apoptosis in the pathogenesis of SS and RA. In RA, FLS are mainly differentiated from mesenchymal stem cells and have a strong proliferative and invasive tendency similar to tumor cells. Their behavior is crucial in pannus development and bone destruction, and they can also prevent apoptosis^11^. In SS, increased apoptosis of ductal epithelial cells and acinar cells of the salivary glands results in atrophy and gland function loss^5^. As far we know, this is the first study to report that IGU can improve glandular exocrine function by inhibiting apoptosis in exocrine gland epithelial cells; however, the relationship between apoptosis and IGU remains to be further studied.

This study has certain limitations. NOD mouse is a genetically related mouse model, which is different from human innate and adaptive immunity. A single mouse model does not recapitulate all disease features of human SS and only represents a subset of patients. Data obtained from NOD mouse models should be carefully evaluated and interpreted based on knowledge of the mouse genetic background and further confirmed in conjunction with clinical studies. This experiment only observed the changes of some inflammatory factors and apoptosis-related proteins through animal experiments, which cannot fully clarify the mechanism of action of iguratimod on NOD mice. It is not possible to set a higher dose group to further investigate whether the efficacy is dose-dependent. In addition, the toxicity and excretion of IGU could not be monitored.

## Conclusions

IGU might attenuate the expression of inflammatory factors in the serum and regulate the expression of apoptosis-associated proteins in exocrine glands. It can mitigate the infiltration of exocrine gland lymphocytes and decrease the extent of apoptosis in exocrine gland epithelial cells, thereby improving exocrine gland secretory function in model mice.

### Supplementary Information

Supplementary Information 1.

Supplementary Information 2.

## Data Availability

All data generated or analysed during this study are included in this published article and its supplementary information files. The datasets generated and analysed during the current study are not publicly available, but are available from the corresponding author on reasonable request.
