# Supplementary material for: Effects of iguratimod on inflammatory factors and apoptosis of submandibular gland epithelial cells in NOD mice
Source: Sci Rep. 2023 Oct 24;13:18205. doi: 10.1038/s41598-023-45529-x (PMC10597989; doi:10.1038/s41598-023-45529-x)
Supplement: Supplementary file 1 — Supplementary Information 1. [file 41598_2023_45529_MOESM1_ESM.pdf]

Water intak of mice in each group of the 1w (ml)																
	W0	M1							water intake for each group (n=8)							Average per day
		1d	2d	3d	4d	5d	6d	7d	1d	2d	3d	4d	5d	6d	7d	
Normal	250	219	222	214	221	221	218	221	31	28	36	29	29	32	29	30.83
Model	250	208	209	210	206	207	213	208	42	41	40	44	43	37	42	41.29
IGU 10mg/kg	250	208	206	209	207	209	208	210	42	44	41	43	41	42	40	41.86
IGU 30mg/kg	250	207	206	211	210	208	212	210	43	44	39	40	42	38	40	40.86

Water intake of mice in each group of the 2w (ml)																
	M0	M1							water intake for each group (n=8)							Average per day
		1d	2d	3d	4d	5d	6d	7d	1d	2d	3d	4d	5d	6d	7d	
Normal	250	218	215	217	218	216	220	218	32	35	33	32	34	30	32	32.57
Model	250	210	201	203	203	204	201	203	40	49	47	47	46	49	47	46.43
IGU 10mg/kg	250	209	210	206	208	207	210	209	41	40	44	42	43	40	41	41.57
IGU 30mg/kg	250	210	209	208	209	207	207	211	40	41	42	41	43	43	39	41.29

Water intake of mice in each group of the 3w (ml)																
	M0	M1							water intake for each group (n=8)							Average per day
		1d	2d	3d	4d	5d	6d	7d	1d	2d	3d	4d	5d	6d	7d	
Normal	250	211	217	219	219	218	221	214	39	33	31	31	32	29	36	33.00
Model	250	203	205	203	204	201	199	199	47	45	47	46	49	51	51	48.00
IGU 10mg/kg	250	207	204	206	206	211	205	207	43	46	44	44	39	45	43	43.43
IGU 30mg/kg	250	210	205	208	209	210	205	209	40	45	42	41	40	45	41	42.00

Water intake of mice in each group of the 4w (ml)																
	M0	M1							water intake for each group (n=8)							Average per day
		1d	2d	3d	4d	5d	6d	7d	1d	2d	3d	4d	5d	6d	7d	
Normal	250	216	214	216	215	217	215	210	34	36	34	35	33	35	40	35.29
Model	250	201	200	200	198	198	197	197	49	50	50	52	52	53	53	51.29
IGU 10mg/kg	250	209	210	210	203	201	202	203	41	40	40	47	49	48	47	44.57
IGU 30mg/kg	250	210	210	209	205	203	203	208	40	40	41	45	47	47	42	43.14

Water intake of mice in each group of the 5w （ml）																
	M0	M1							water intake for each group (n=8)							Average per day
		1d	2d	3d	4d	5d	6d	7d	1d	2d	3d	4d	5d	6d	7d	
Normal	250	233	212	212	227	213	215	215	17	38	38	23	37	35	35	36.60
Model	250	228	196	195	198	221	195	193	22	54	55	52	29	55	57	54.60
IGU 10mg/kg	250	203	202	204	204	204	203	201	47	48	46	46	46	47	49	47.00
IGU 30mg/kg	250	203	202	204	205	205	206	203	47	48	46	45	45	44	47	46.00

Water intake of mice in each group of the 6w (ml)																
	M0	M1							water intake for each group (n=8)							Average per day
		1d	2d	3d	4d	5d	6d	7d	1d	2d	3d	4d	5d	6d	7d	
Normal	250	213	212	213	214	213	212	214	37	38	37	36	37	38	36	37.00
Model	250	194	194	195	194	204	194	192	56	56	55	56	46	56	58	56.17

IGU 10mg/kg	250	203	207	202	204	200	198	201	47	43	48	46	50	52	49	47.86
IGU 30mg/kg	250	207	205	205	205	206	202	205	43	45	45	45	44	48	45	45.00
Water intake of mice in each group of the 7w (ml)																
	M0	M1							water intake for each group (n=8)							Average per day
		1d	2d	3d	4d	5d	6d	7d	1d	2d	3d	4d	5d	6d	7d	
Normal	250	213	215	214	214	214	215	212	37	35	36	36	36	35	38	36.14
Model	250	192	192	194	193	196	193	188	58	58	56	57	54	57	62	57.43
IGU 10mg/kg	250	204	201	202	204	203	204	205	46	49	48	46	47	46	45	46.71
IGU 30mg/kg	250	206	207	204	205	207	206	206	44	43	46	45	43	44	44	44.14
Water intake of mice in each group of the 8w (ml)																
	M0	M1							water intake for each group (n=8)							Average per day
		1d	2d	3d	4d	5d	6d	7d	1d	2d	3d	4d	5d	6d	7d	
Normal	250	214	213	215	212	216	214	213	36	37	35	38	34	36	37	36.00
Model	250	188	186	195	194	192	192	195	62	64	55	56	58	58	55	58.83
IGU 10mg/kg	250	203	204	205	203	203	204	203	47	46	45	47	47	46	47	46.33
IGU 30mg/kg	250	205	206	203	204	207	206	205	45	44	47	46	43	44	45	44.83

W0: water given for mice daily at 9:00 am

W1: the remaining water at 9:00am the next day

Data with a red background were discarded due to a large gap with other data
