# Supplementary material for: Effects of iguratimod on inflammatory factors and apoptosis of submandibular gland epithelial cells in NOD mice
Source: Sci Rep. 2023 Oct 24;13:18205. doi: 10.1038/s41598-023-45529-x (PMC10597989; doi:10.1038/s41598-023-45529-x)
Supplement: Supplementary file 2 — Supplementary Information 2. [file 41598_2023_45529_MOESM2_ESM.pdf]

Western blotting

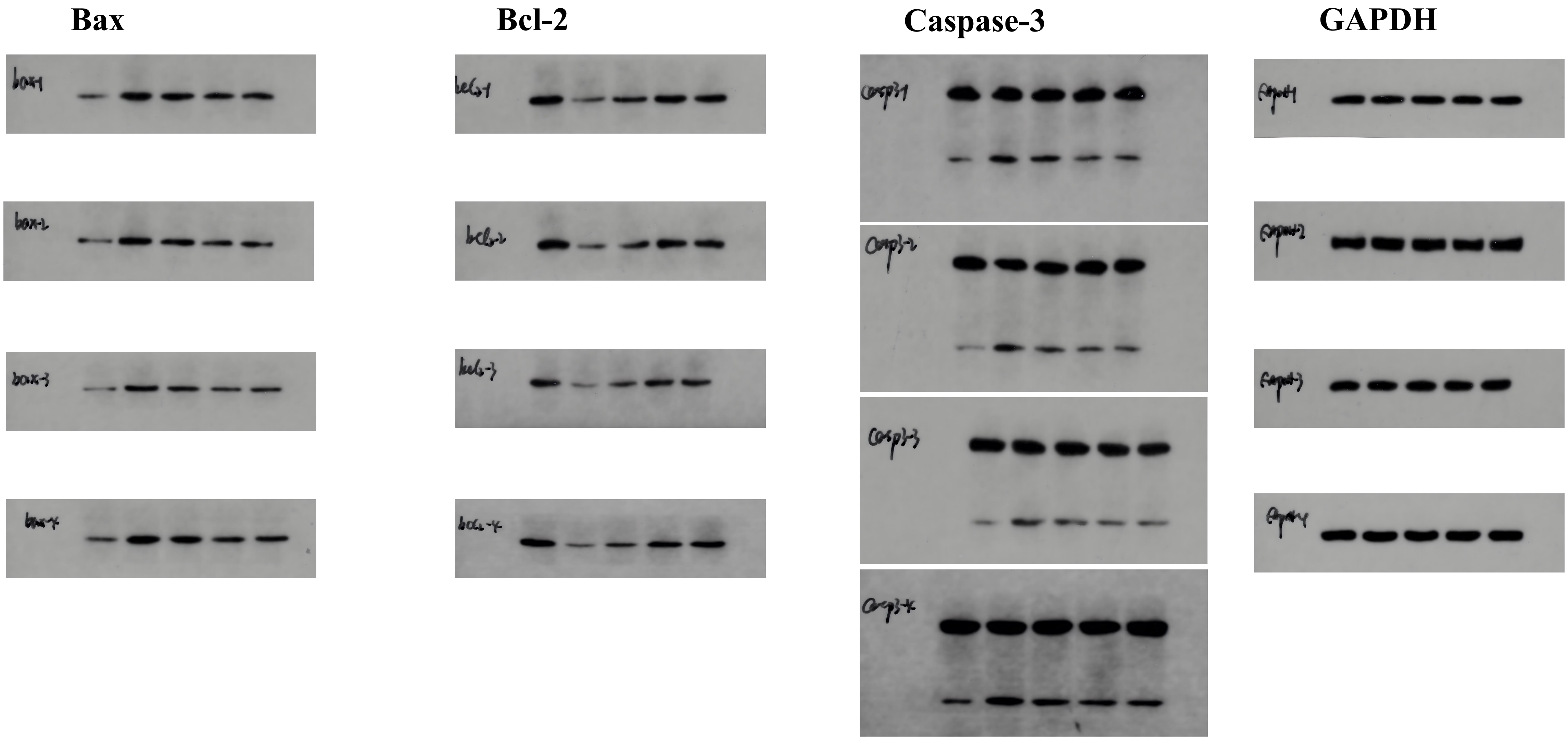

There are 5 groups in the picture, as normal group, model group, IGU 10mg/kg, IGU 30mg/kg, the last group is the Chinese medicine group, and the relevant experiments related to the Chinese medicine group are introduced in another article.

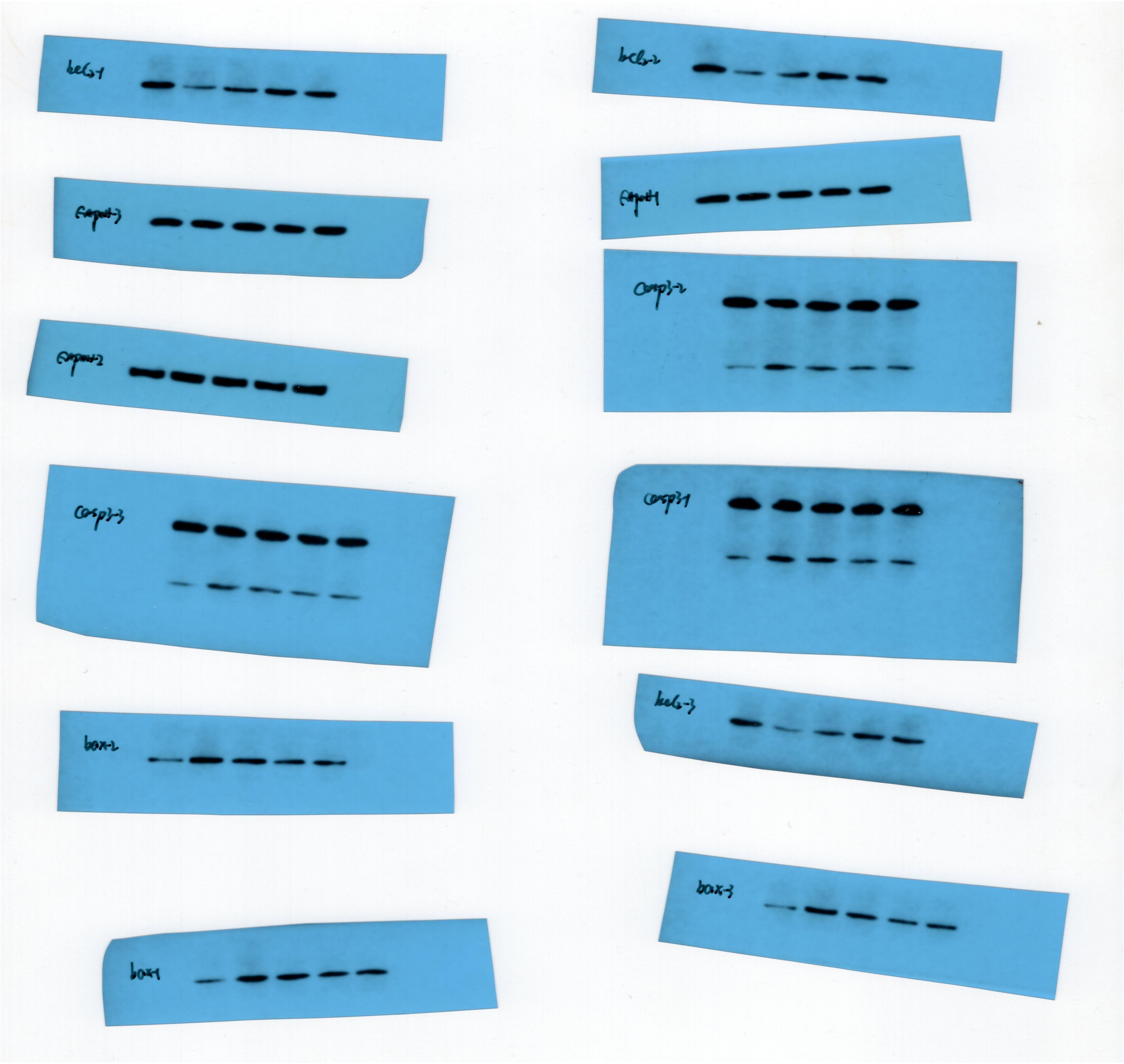

There are 5 groups in the picture, as normal group, model group, IGU 10mg/kg, IGU 30mg/kg, the last group is the Chinese medicine group, and the relevant experiments related to the Chinese medicine group are introduced in another article.
